# *Bifidobacterium*: a probiotic for the prevention and treatment of depression

**DOI:** 10.3389/fmicb.2023.1174800

**Published:** 2023-05-10

**Authors:** Jiayu Li, Junyu Wang, Meiyu Wang, Li Zheng, Qiuyu Cen, Fangfang Wang, Li Zhu, Rizhao Pang, Anren Zhang

**Affiliations:** ^1^School of Health Preservation and Rehabilitation, Chengdu University of Traditional Chinese Medicine, Chengdu, China; ^2^Department of Rehabilitation Medicine, General Hospital of Western Theater Command, Chengdu, China; ^3^State Key Laboratory of Biotherapy, West China Hospital, Sichuan University, Chengdu, China; ^4^Rehabilitation and Wellness Care Centre, Tianfu College of Swufe, Chengdu, China; ^5^Department of Rehabilitation Medicine, Shanghai Fourth People’s Hospital Affiliated to Tongji University School of Medicine, Shanghai, China

**Keywords:** *Bifidobacterium*, depression, gut microbiota, probiotics, mechanism

## Abstract

Depression is a common psychological disease, which has become one of the main factors affecting human health. It has a serious impact on individuals, families, and society. With the prevalence of COVID-19, the incidence of depression has further increased worldwide. It has been confirmed that probiotics play a role in preventing and treating depression. Especially, *Bifidobacterium* is the most widely used probiotic and has positive effects on the treatment of depression. The mechanisms underlying its antidepressant effects might include anti-inflammation and regulation of tryptophan metabolism, 5-hydroxytryptamine synthesis, and the hypothalamus-pituitary–adrenal axis. In this mini-review, the relationship between *Bifidobacterium* and depression was summarized. It is hoped that *Bifidobacterium*-related preparations would play a positive role in the prevention and treatment of depression in the future.

## 1. Introduction

Depression is a heterogeneous mental disorder, which seriously affects the daily life of patients. The clinical symptoms (such as feelings of sadness or emptiness) of depression may last for months, years, or even the whole life. Studies have shown that depressive symptoms are involved in the occurrence and development of various diseases, such as diabetes (Stuart and Baune, 2012; Moulton et al., 2015), cardiovascular diseases (Vance et al., 2019; Bucciarelli et al., 2020), and obesity (Mannan et al., 2016a,b). Some patients with severe depression may even have suicidal behaviors (Ribeiro et al., 2018; Liu et al., 2022). According to the statistics of the World Health Organization, about 1 billion people worldwide are suffering from mental disorders (World Health Organization, 2022). During the COVID-19 epidemic, the number of people suffering from depression and anxiety has increased by more than 25% globally, and mental health has become one of the main factors causing the global burden of disease (World Health Organization, 2023). The pressure of epidemic prevention and control has brought serious challenges to the diagnosis and treatment of depression. However, the pathogenesis of depression remains unclear. The etiology of depression may be the result of the interaction between multiple factors, including genetic factors, environmental factors, and body heterogeneity. Pan et al. (2022) have the OGDHL (oxoglutarate dehydrogenase) rs2293239 (p.Asn725Ser) as one of the main genetic factors for family depression through multigroup analysis. The insulting experiences from social peers and childhood traumatic experiences are high-risk factors for the occurrence of depression (Wang et al., 2023). The introduction of effective environmental interventions such as green vegetation and water sources into living environments such as campuses and communities have become potential protective factors against depression (Yang et al., 2022). In addition, female gender, low income, poor parental relationships, singleness, smoking and alcohol abuse, dietary structure, gut microbiota, and other heterogeneous factors have been reported to be related to depression (Milaneschi et al., 2011; van Sprang et al., 2023). Although the cure rate of depression treatment has improved during the past 40 years, the prevalence rate has not declined (Ormel et al., 2019). It is worth noting that the age of depression cases has become younger. It is shown that the prevalence of depression among adolescents aged 10–24 years has increased sharply in the past decade, and women are more vulnerable to depression (Collishaw, 2015). Moreover, the susceptibility of female adolescents seems to increase as they grow up (Oldehinkel and Bouma, 2011; Platt et al., 2021).

Studies have shown that adult mental disorders originate from the neurodevelopmental stage of early life and peak in middle and late adolescence (Howes and Murray, 2014; Gałecki and Talarowska, 2018). Adolescence is a period of critical transformation in life. The mental disorders that occur during this period would cause extensive and lasting damage in future interpersonal communication, education, and occupation. Based on the evidence that the age of patients with depression has become younger, early detection and prevention of this mental disease becomes increasingly important. Meanwhile, it is necessary to develop new treatment methods to solve this medical problem.

Because of their key role in the regulation of the central nervous system, the gut microbiota is called the human “second brain” (Ridaura and Belkaid, 2015). More and more studies have shown that there is a gut-brain axis between intestinal microorganisms and the central nervous system (Rutsch et al., 2020; Doifode et al., 2021). Many mechanisms are involved in the gut-brain axis, including the vagus nerve, microbial metabolism, hormones, and immune regulation, thus affecting cognitive function and emotion (Heijtz et al., 2011; Foster and McVey Neufeld, 2013; Sandhu et al., 2017). Several studies have found that gut microbiota can affect brain function to improve depressive symptoms (Bravo et al., 2011; Hsiao et al., 2013).

The gut microbiota of depressed patients is disordered and is significantly different from that of healthy people (Barandouzi et al., 2020). The gut microbiota disorder has also been shown in animal models (Wong et al., 2016). Targeted regulation of gut microbiota has become a new strategy for the prevention and treatment of depression. At present, the probiotic preparations of *Bifidobacterium* and *Lactobacillus* have been commonly used to improve the symptoms of depression (Ng et al., 2018). Among them, the *Bifidobacterium* preparation has been confirmed to have a positive impact on the improvement of depression symptoms, and the relevant products have been used as effective adjunctive agents for the clinical treatment of depression. For example, the patients with mild to moderate depressive symptoms accompanied by irritable bowel syndrome (IBS) who were treated with *Bifidobacterium longum* NCC3001 had significantly improved depressive symptoms and quality of life scores compared with the placebo group, as well as improved responses to multiple brain regions (including the amygdala and the marginal frontal lobe) to negative emotional stimuli (Pinto-Sanchez et al., 2017).

In this mini-review, the current knowledge about the relationship between *Bifidobacterium* and depression has been introduced, including the relationship between depression and *Bifidobacterium*, and the possible antidepressant mechanism. In addition, the research progress in the treatment of depression with *Bifidobacterium* preparations or *Bifidobacterium* combination preparations in use and development has also been summarized (Table 1). Finally, we emphasize that the *Bifidobacterium*-related drug preparations may be used as a new adjuvant therapy to prevent the occurrence and development of depression.

**Table 1:** Summary of the application of *Bifidobacterium*-related preparations in the intervention of depression.

*Bifidobacterium* related preparation	Experimental model	Content	Duration of intervention	Results
*Lactobacillus helveticus* R0052 *Bifidobacterium longum* R0175 (Arseneault-Bréard et al., 2012)	Sprague–Dawley rats	1.0 × 10^9^ live bacterial cells	2 weeks	Intervened the development of post-MI depressive behavior Reduced IL-1β level Maintained intestinal barrier integrity
*Bifidobacteria infantis* 35624 (Desbonnet et al., 2010)	Sprague–Dawley rats	1 × 10^10^ live bacterial cells	2 weeks	Reduced swim behavior and increased immobility in the FST Restored noradrenaline concentrations Attenuated the exaggerated IL-6 response
*Bifidobacteria infantis* 35624 (Desbonnet et al., 2008)	Sprague–Dawley rats	1 × 10^10^ live bacterial cells	2 weeks	Reduced 5-HIAA concentration Elevated the serotonergic precursor, tryptophan Attenuated IFN-γ, TNF-α and IL-6 cytokines Decreased DOPAC in the amygdaloid cortex
*Lactobacillus reuteri* NK33 *Bifidobacterium adolescentis* NK98 (Han et al., 2020)	C57BL/6 mice	1 × 10^9^ CFU	5 days	Increased BDNF^+^/NeuN^+^ cell population Suppressed NF-κB action in the hippocampus Alleviated gut dysbiosis
*Lactobacillus reuteri* NK33 *Bifidobacterium adolescentis* NK98 (Jang et al., 2019)	C57BL/6 mice	1 × 10^9^ CFU	5 days	Suppressed depressive behavior Suppressrf NF-κB activation in lipopolysaccharide (LPS) Suppressed the infiltration of Iba1^+^ and LPS^+^/CD11b^+^ cells Suppressed corticosterone, IL-6, and LPS levels in the blood Induced hippocampal BDNF expression Suppressed the IS-induced fecal proteobacteria population
*Bifidobacterium breve* M-16 V (Kosuge et al., 2021)	C57BL/6J mice	5.0 × 10^9^ nonviable cells	33 days	Prevented social interaction impairment Suppressed IL-1β increase in the prefrontal cortex and hippocampus Modulated the gut microbiota composition
*Bifidobacterium longum subsp. infantis* E41 *Bifidobacterium breve* M2CF22M7 (Tian et al., 2019)	C57BL/6J mice	1 × 10^9^ CFU	5 weeks	Reduced depressive behaviors of mice in the forced swim test, sucrose preference test, and step-down test Improved the expression of Tph1 and secretion of 5-HTP in RIN14B cells Increased the level of 5-HTP and brain-derived neurotrophic factor concentration in the brain Reduced the serum corticosterone level Improved microbial dysbiosis
*Bifidobacterium bifidum Lactobacillus acidophilus Lactobacillus casei* (Akkasheh et al., 2016)	MDD patients	2 × 10^9^ CFU	8 weeks	Decreased BDI total score Decreased serum insulin levels and serum hs-CRP concentrations Improved plasma total glutathione levels
*Lactobacillus reuteri* NK33 *Bifidobacterium adolescentis* NK98 (Lee et al., 2021)	Healthy adults with subclinical symptoms of depression, anxiety, and insomnia	2.0 × 10^9^ CFU	8 weeks	Decreased the BAI score and BDI-II score Decreased serum IL-6 levels Restored gut microbiota composition
*Bifidobacterium breve* A-1 (Okubo et al., 2019)	Patients with schizophrenia	5 × 10^10^ CFU	4 weeks	25% reduction in Hospital Anxiety and Depression Scale (HADS) total score Improved Positive and Negative Syndrome Scale (PANSS) anxiety/depression score Increased IL-22 and TRANCE expression
*Bifidobacterium longum* NCC3001 (Pinto-Sanchez et al., 2017)	Patients with IBS and diarrhea or a mixed-stool pattern	1 × 10^10^ CFU	6 weeks	Reduced depression scores of 2 points or more on HAD-D scores Reduced responses to negative emotional stimuli in multiple brain areas Reduced urine levels of methylamines and aromatic amino acids metabolites
*Bifidobacterium breve* CCFM1025 (Tian et al., 2022)	MDD patients	1 × 10^10^ CFU	4 weeks	Decreased Hamilton Depression Rating scale-24 (HDRS-24) rating score Reduced the 5-HT turnover Changed the tryptophan metabolism of the gut microbiome Increased level of tryptophan, 5-HTP, 5-HT
*Bifidobacterium bifidum* W23 *Bifidobacterium lactis* W52 *Lactobacillus acidophilus* W37 *Lactobacillus brevis* W63 *Lactobacillus casei* W56 *Lactobacillus salivarius* W24 *Lactococcus lactis* (W19 and W58) (Steenbergen et al., 2015)	Healthy adults	5 × 10^9^ CFU	4 weeks	Reduced overall cognitive reactivity to depression and in particular aggressive and ruminative thoughts

## 2. Depression and gut microbiota

Gut microbiota is composed of 10^14^ to 10^15^ bacterial cells, and distributed in more than 1000 species (Li et al., 2014). There is a strong correlation between depression and the imbalance of gut microbiota. A previous study has found that the abundance of *Bifidobacteria* and *Lactobacilli* in patients with major depressive disorder (MDD) was significantly decreased (Aizawa et al., 2016). Another study has found that the abundance of *Bacteroides*, *Proteobacteria*, and *Actinomycetes* in MDD patients was significantly increased, while the abundance of *Bacterobacter* was significantly decreased. Compared with the control group, the abundance of *Lachnospiraceae* and *Ruminococcaceae* families, within the phylum Firmicutes, is decreased in the A-MDD group (HAMDS score ≥ 20) (Jiang et al., 2015). In addition, although most studies reported that the bacteria in patients with depression belong to *Chlamydomonas* at the family and genus level, there is no consensus on the abundance of *Chlamydomonas* (Barandouzi et al., 2020). Although the change of gut microbiota in patients with depression may lead to different results due to individual differences, the disorder of gut microbiota is certain.

The microbiota of MDD patients have been transplanted into germ-free mice, which then showed depressive behavior (Zheng et al., 2016). In addition, the density of microglia in the ventral hippocampus of healthy rats was increased after treatment with the microbiota of rats with depressive behavior, and the expression of interleukin-1β (IL-1β) was also increased, showing obvious depressive behavior compared with the control group (Pearson-Leary et al., 2020). Similarly, transplanting the fecal microbiota of patients with depression to rats lacking microbiota can also induce depression and anxiety-like behavior in recipient animals, and alter the tryptophan metabolism (Kelly et al., 2016). Studies have shown that fecal microbiota transplantation (FMT) can improve the depressive symptoms of patients with IBS, functional diarrhea, and functional constipation (Kurokawa et al., 2018; Lin et al., 2021). Oral administration of FMT capsules can improve the depression and anxiety scores of IBS patients complicated with diarrhea and psychological disorders (Guo et al., 2021). In addition, FMT can improve stool patterns and depressive symptoms by increasing the diversity of gut microbiota in IBS patients (Mizuno et al., 2017). The above evidence indicates that the gut microbiota is closely related to the occurrence and development of depression. Based on existing evidence, improving depressive symptoms by regulating the gut microbiota may provide a new idea for the prevention and treatment of depression. However, due to the complex pathogenesis of depression, whether the imbalance of gut microbiota is the cause of depression remains to be further explored.

## 3. Biological characteristics of *Bifidobacterium*

In 2001, scientists from the Food and Agriculture Organization and the World Health Organization of the United Nations revised the definition of probiotics at an expert consultation as follows: when given at a sufficient amount, probiotics will be beneficial to the health of the host. Since then, this definition has been most widely accepted in the world (Hill et al., 2014). Studies have confirmed that probiotics play a certain role in the treatment of IBS (Ford et al., 2014; Didari et al., 2015), inflammatory bowel disease (Hegazy and El-Bedewy, 2010; Park et al., 2022), constipation (Barichella et al., 2016; Tan et al., 2020), respiratory infection (Li et al., 2019; Mai et al., 2021), and emotional disorders (Steenbergen et al., 2015; Tran et al., 2019). It is noteworthy that *Bifidobacteria* have beneficial effects on depression (Dinan et al., 2013; Aizawa et al., 2016).

*Bifidobacterium* is a typical intestinal bacterium, belonging to *Actinobacillus* and *Bifidobacteriaceae*. It is a kind of inactive, spore-producing, and gas-producing Gram-positive bacterium (Bottacini et al., 2014). It is considered to be the most important bacterial group in the intestinal microbial community of vaginal delivery and breastfed infants (Turroni et al., 2012). *Bifidobacteria* are the first batch of microbial colonizers in the intestines of newborns, and thus play a key role in their physiological development, including the maturation of the immune system and food catabolism (Hidalgo-Cantabrana et al., 2017). Shortly after birth, up to 90% of the bacteria found in the gastrointestinal tract of infants are *Bifidobacteria* (Harmsen et al., 2000). In adults, they still account for 3–5% of the total intestinal microbial community (Harmsen et al., 2002). Among them, *Bifidobacterium longum*, *Bifidobacterium adolescentis*, *Bifidobacterium breve*, *Bifidobacterium pseudo streptococcus*, and *Bifidobacterium pseudolongum* are dominant, and therefore these species have been considered to be the widely existing *Bifidobacterium* species (Turroni et al., 2009).

## 4. Antidepressant effects and mechanisms of *Bifidobacterium*

**Figure 1:**
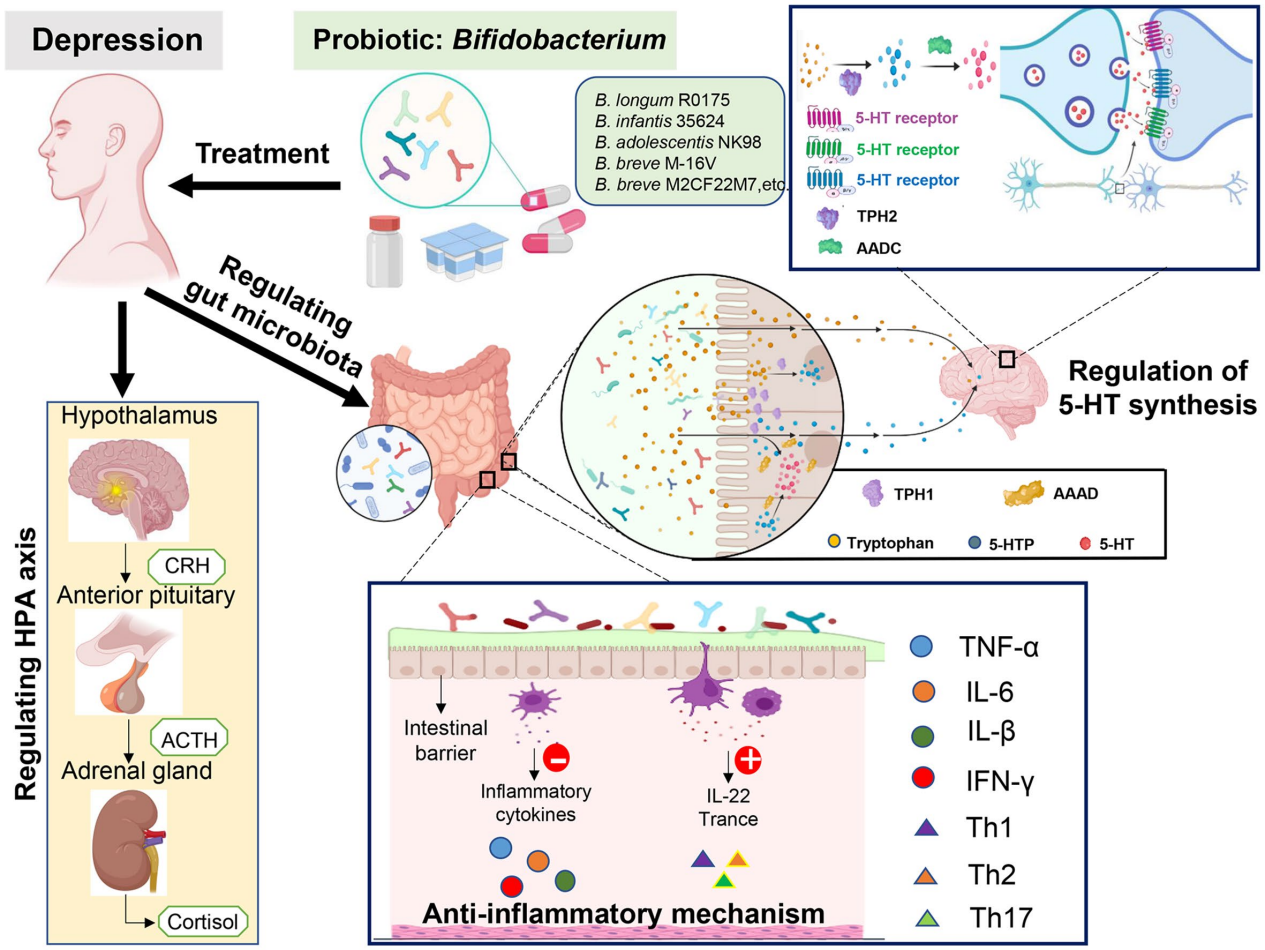
The antidepressant mechanisms of *Bifidobacterium*.

Increasing evidence has shown that probiotics containing *Bifidobacteria* may have the potential to prevent and treat various mental and psychological diseases, such as depression and anxiety (Cheng et al., 2019). Steenbergen et al. (2015) conducted a three-blind randomized placebo-controlled trial, in which healthy subjects were daily treated with lyophilized probiotics powder containing *Bifidobacteria* (2.5 × 10^9^ CFU). Before and after the intervention, the subjects were evaluated with the revised Leiden Depression Sensitivity Scale (LEIDS-r), Becker Depression Scale II (BDI-II), and Becker Anxiety Scale (BAI). The results have shown that the intake of probiotics for 4 weeks would significantly reduce the overall cognitive response to depression, especially aggressive and reflective thinking, and the results first confirmed that, the 4-week intervention of multi-species probiotics had a positive impact on the cognitive response of the natural sadness mood changes in healthy individuals. Other studies have shown that after treatment with *Bifidobacterium infantis*, the maternally isolated rats would show a reversed immune function, reversed norepinephrine concentration in the brain, and finally reversed depressive behavior (Desbonnet et al., 2010). Pinto-Sanchez et al. (2017) used 1.0 × 10^10^ CFU *Bifidobacterium longum* NC3001 to treat adult IBS patients with mild and moderate depression for 6 weeks, and the results have shown that after the intervention, the depression score of the experimental group was significantly lower than the control group. In addition, the results of functional magnetic resonance imaging showed that *Bifidobacterium longum* NC3001 reduced the response of multiple brain regions (mainly including the amygdala and frontal limbic regions) to fear stimuli, and the decreased activation of the amygdala frontal limbic complex was related to the decreased depression score. These findings suggest that *Bifidobacterium* has a positive role in depression treatment. A large number of studies (Han et al., 2020; Tian et al., 2022) have confirmed that *Bifidobacteria* can be used to treat depressive symptoms, but the underlying mechanisms have not been completely elucidated, which may be related to reducing the abundance of pathogenic bacteria, exerting the anti-inflammatory effects, improving the permeability of intestinal barrier, regulating the tryptophan levels, affecting 5-hydroxytryptamine (5-HT) synthesis, and regulating hypothalamus-pituitary–adrenal (HPA) axis (Figure 1).

### 4.1. Anti-inflammatory mechanisms

Inflammation exacerbation is one of the characteristics of a series of diseases, including cardiovascular disease, diabetes, metabolic syndrome, rheumatoid arthritis, asthma, multiple sclerosis, and chronic pain, each of which would increase the risk of depression (Dantzer et al., 2008; Slavich and Irwin, 2014). Cytokines induce depressive symptoms by affecting different emotion-related processes. Elevated inflammatory signals would cause neurotransmitter metabolism disorders, damage nerve health, and disrupt brain regulation and signal mechanisms in behavior and emotion (Johnson et al., 2021). Some studies (Stewart et al., 2009; Matheny et al., 2011; Jin et al., 2020) have shown that compared with individuals with less frequency of depressive symptoms, individuals with more frequency of depressive symptoms would produce more interleukin-6 (IL-6) after being stimulated, showing that the level of IL-6 is significantly increased after stimulation (Fagundes et al., 2013). In the stress model, increased IL-1β in the brain induced depressive behavior, while the IL-1 receptor gene knockout mice did not exhibit depressive behavior after chronic social failure stress (CSDS) exposure (McKim et al., 2018). Mice exposed to CSDS for 10 consecutive days showed changes in intestinal microbial composition and IL-1β expression in the brain and had increased depressive behavior. Intervention with heat-sterilized *Bifidobacterium breve* M-16V could reduce the abundance levels of bacteria to prevent CSDS-induced depression and IL-1β expression (Kosuge et al., 2021). Desbonnet et al. (2008) have pointed out that *Bifidobacterium infantis* 35624 containing 1 × 10^10^ live bacterial cells can reduce the proinflammatory cytokines IL-6, IFN-γ, and TNF-α in SD rats after treatment for 14 days. In a randomized, double-blind, placebo-controlled parallel study, people with subclinical depressive symptoms were given the probiotics NVP-1704 containing 0.5 × 10^9^ CFU *Bifidobacterium adolescentis* NK98, and after treatment, the depressive symptoms of this group were significantly improved, with significantly reduced serum level of IL-6. Human gut microbiota is closely related to the production of pro-inflammatory cytokines (such as IL-6).

Depression can promote intestinal permeability, that is, greater inflammation induces endotoxin translocation, which could be described as *intestinal leakage* (Maes et al., 2008). Exposure to pro-inflammatory cytokines such as TNF-α will reduce the function of the epithelial barrier, which may lead to the translocation of bacterial components into the blood, and the permeability of the intestinal barrier may be involved (Arseneault-Bréard et al., 2012). The intestinal epithelium forms a barrier between the external environment and lumen contents, in which the tight junction complex plays an important regulatory role (Cario, 2008). This ensures the impermeability of the intestinal barrier and effectively avoids the translocation of intraluminal antigens, toxins, and inflammatory compounds (Goyer et al., 2016; Paradis et al., 2021). It has been reported that intervention with *Bifidobacterium infantis* conditioned medium in mice significantly reduced their colon permeability, while the long-term use alleviated the inflammatory response in IL-10 deficient mice, restored colon permeability to a normal level, and reduced the secretion of interferon in the colon (Ewaschuk et al., 2008). The imbalance of gut microbiota, such as the decrease of *Bifidobacterium* and *Lactobacillus,* and the increase of pathogenic bacteria, can stimulate the secretion of pro-inflammatory cytokines by increasing the intestinal epithelial permeability, while *Lactobacillus* and *Bifidobacterium* can down-regulate the secretion of pro-inflammatory cytokines. Therefore, inhibiting the secretion levels of IL-6 may be the reason why NVP-1704 plays a therapeutic role (Lee et al., 2021). The combination of *Bifidobacterium longum* R0175 and *Lactobacillus helveticus* R0052 can regulate depressive behavior after myocardial infarction in rats, including loss of pleasure (the sucrose preference test) and behavioral despair (the forced swimming test), and significantly reduce plasma IL-1β concentration, restoring the integrity of rat intestinal barrier (Arseneault-Bréard et al., 2012). Okubo et al. (2019) have shown that after treatment with 5.0 × 10^10^ *Bifidobacterium breve* A-1 for 4 consecutive weeks, the total scores of the anxiety and depression scale in schizophrenic patients were significantly improved than the baseline. Moreover, the expression levels of IL-22 and TNF-related activation-induced cytokines, which play key roles in the intestinal epithelial barrier function, were significantly up-regulated. The authors speculate that the effect of *Bifidobacterium breve* A-1 on anxiety and depression may be related to the enhancement of intestinal epithelial barrier function. The above findings suggest that *Bifidobacterium* may have antidepressant effects by reducing the secretion of inflammatory factors and restoring the function of the intestinal epithelial barrier.

### 4.2. Regulating tryptophan levels and affecting 5-HT synthesis

The 5-HT is a widely studied neurotransmitter widely distributed in the nervous system and is responsible for a variety of physiological processes, such as mood, sleep, intestinal movement, and vasoconstriction (Chen et al., 2021). There is a close relationship between 5-HT and depression (Boku et al., 2018), but it is still unclear whether dysfunction in the synthesis and release of 5-HT is a direct cause of depression. Recently, Erritzoe et al. reported that the 5-HT release was decreased in patients with the major depressive syndrome, which provides clear evidence for the presence of 5-HT neurotransmission dysfunction in patients with depression (Erritzoe et al., 2022). In a mouse model of stress-induced depression, the levels of 5-HT in the prefrontal cortex and hippocampus were downregulated (Zhang et al., 2018). After intervention with antidepressants, the depressive behaviors of mild stress mice (Li et al., 2018) and postpartum depression rats (Jia et al., 2017) would be significantly alleviated, accompanied by a reversal of the downregulation of 5-HT levels in the prefrontal cortex. Thus, the synthesis and release of 5-HT play important roles in the occurrence and development of depression.

Tryptophan, an essential amino acid obtained from food, is a precursor for 5-HT biosynthesis *in vivo*, and therefore the level of tryptophan directly affects the synthesis of 5-HT (Alcaino et al., 2018). More than 90% of 5-HT is synthesized in the intestinal tract, where tryptophan is first converted into 5-hydroxytryptophan under the action of tryptophan hydroxylase 1 (TPH1), and then into 5-HT under the action of aromatic amino acid decarboxylase (Visser et al., 2011; Maffei, 2020). The tryptophan and 5-Hydroxy-l-tryptophan (5-HTP) in the gut can pass through the blood–brain barrier and enter the brain. Tryptophan hydroxylase 2 expressed in neurons in the raphe nucleus of the brain stem can convert tryptophan into 5-HTP. The 5-HTP can be converted into 5-HT under the action of aromatic L-amino acid decarboxylase. Clinically, the plasma tryptophan level in patients with depression is decreased (Messaoud et al., 2019), and supplementation with tryptophan can improve their depressive symptoms (Gonzalez et al., 2021). Interestingly, the intestinal microbiota can regulate 5-HT by changing the level of tryptophan. For example, *Bifidobacterium longum* infant E41 and *Bifidobacterium breve* M2CF22M7 can improve the microbial imbalance induced by chronic unpredictable mild stress in mice, and regulate the expression of TPH1 in RIN14B cells and the secretion of 5-HTP, significantly reducing the depressive behavior of mice in the forced swimming tests, sucrose preference tests, and hypotensive tests (Tian et al., 2019). The levels of metabolites (including tryptophan, 5-HTP, and 5-HT) in the olfactory bulb of chronic mill stress mice show a downward trend (Chen et al., 2022). *Bifidobacterium* can achieve antidepressant effects by regulating tryptophan. *Bifidobacterium breve* CCFM1025 has been shown to exert antidepressant effects by upregulating related substances including tryptophan, 5-HTP, and 5-HT (Chen et al., 2022). Taken together, *Bifidobacteria* may affect the synthesis of 5-HT by regulating the level of tryptophan.

### 4.3. Regulating the hypothalamus-pituitary–adrenal (HPA) axis

The change of the HPA axis in depression may reflect the influence of stress and regulate the performance of depressive symptoms (Peng et al., 2015). Stress activates the HPA axis and finally stimulates the adrenal cortex to release glucocorticoids (i.e., human cortisol and animal cortisol) in response to the stimulation of adrenocorticotropic hormone from the anterior pituitary. The increased glucocorticoids in circulation inhibit the secretion of corticotropin-releasing hormone (CRH) and vasopressin in the hypothalamus, establishing a negative feedback loop. On the other hand, stress-induced intestinal ecological imbalance aggravates intestinal inflammation and permeability and stimulates the release of pro-inflammatory cytokines, thus further activating the HPA axis (Johnson et al., 2021). The intervention of the probiotics mixture containing *Bifidobacterium* W23 in high-fat diet model rats can significantly reduce the depressive behavior in the forced swimming test, and reduce the transcription level of factors involved in the regulation of the HPA axis (CRH-R1, CRH-R2, and MR) in the hippocampus. Therefore, the antidepressant effects of *Bifidobacterium* may be affected by the HPA axis (Abildgaard et al., 2017). In the mouse model suffering from chronic stress, the combination of *Bifidobacterium longum* R0175 and *Lactobacillus* R0052 would significantly improve depressive behavior, reduce the level of corticosterone, and restore the intestinal barrier. The decrease in cortisol level is related to the reduction of HPA axis hyperactivity (Ait-Belgnaoui et al., 2014). The above findings show that *Bifidobacterium* can improve depressive behavior by regulating the HPA axis.

## 5. Conclusion and future directions

Based on the above evidence, we conclude that *Bifidobacterium* has certain antidepressant effects. However, there are certain differences in the clinical research results, which may be caused by different basic diseases of the study participants and differences in research programs. Depression has a serious impact on the health and quality of life of patients, and effective interventions are urgently needed for disease prevention and treatment. These interventions should be of low cost and have low side effects. In recent years, the intervention with probiotics has been confirmed to regulate intestinal micro-ecology and further improve the disease symptoms, suggesting the potential of probiotic-customized products as a new treatment strategy. This mini-review summarized the evidence of *Bifidobacterium* in the treatment of depression in recent years. *Bifidobacterium*, as a kind of probiotic, could bring positive prevention and treatment efficacy for depression in both model animals and human beings. *Bifidobacterium* can exert its antidepressant role through the anti-inflammatory effects and regulating tryptophan metabolism, 5-HT synthesis, and the HPA axis. Our findings may provide evidence for *Bifidobacterium*-related drug preparations as adjuvant therapy for the prevention and treatment of depression. In the future, the biological mechanism of the antidepressant effects of *Bifidobacterium* still needs to be further clarified.

## Author contributions

AZ, RP, and JW conceived the manuscript. JL, JW, and MW wrote the manuscript. RP, JL, JW, MW, Li Zheng, QC, FW, and Li Zhu contributed to the literature search and provided insightful suggestions in revising this work. All authors contributed to the article and approved the submitted version.

## Funding

This work was supported by the General Program of National Natural Science Foundation of China (81973927), the Key R&D Program of Sichuan Department of Science and Technology (2021YFS0133), the Special Project for Scientific Research of Traditional Chinese Medicine in Sichuan Province (2020), the Special Project Of Traditional Chinese Medicine Scientific Research of Sichuan Provincial Administration of Traditional Chinese Medicine (2020LC0224), the Hospital Project of the General Hospital of the Western Theater (2019), and the Hospital Plateau Medicine Research Project in 2019 (2019ZY03).

## Conflict of interest

The authors declare that the research was conducted in the absence of any commercial or financial relationships that could be construed as a potential conflict of interest.

## Publisher’s note

All claims expressed in this article are solely those of the authors and do not necessarily represent those of their affiliated organizations, or those of the publisher, the editors and the reviewers. Any product that may be evaluated in this article, or claim that may be made by its manufacturer, is not guaranteed or endorsed by the publisher.
